# Loss of Central Inhibition: Implications for Behavioral Hypersensitivity after Contusive Spinal Cord Injury in Rats

**DOI:** 10.1155/2014/178278

**Published:** 2014-08-10

**Authors:** Yerko A. Berrocal, Vania W. Almeida, Rocio Puentes, Eric P. Knott, Jaclyn F. Hechtman, Mary Garland, Damien D. Pearse

**Affiliations:** ^1^Department of Cellular Biology and Pharmacology, Herbert Wertheim College of Medicine, Florida International University, 11200 SW 8th Street, AHC2-481, Miami, FL 33199, USA; ^2^The Miami Project to Cure Paralysis, The University of Miami Miller School of Medicine, Miami, FL 33136, USA; ^3^The Department of Neurological Surgery, The Neuroscience Program, The Interdisciplinary Stem Cell Institute, The University of Miami Miller School of Medicine, Miami, FL 33136, USA

## Abstract

Behavioral hypersensitivity is common following spinal cord injury (SCI), producing significant discomfort and often developing into chronic pain syndromes. While the mechanisms underlying the development of behavioral hypersensitivity after SCI are poorly understood, previous studies of SCI contusion have shown an increase in amino acids, namely, aspartate and glutamate, along with a decrease in GABA and glycine, particularly below the injury. The current study sought to identify alterations in key enzymes and receptors involved in mediating central inhibition via GABA and glycine after a clinically-relevant contusion SCI model. Following thoracic (T8) 25.0 mm NYU contusion SCI in rodents, significant and persistent behavioral hypersensitivity developed as evidenced by cutaneous allodynia and thermal hyperalgesia. Biochemical analyses confirmed upregulation of glutamate receptor GluR3 with downregulation of the GABA synthesizing enzyme (GAD_65/67_) and the glycine receptor *α*3 (GLRA3), notably below the injury. Combined, these changes result in the disinhibition of excitatory impulses and contribute to behavioral hyperexcitability. This study demonstrates a loss of central inhibition and the development of behavioral hypersensitivity in a contusive SCI paradigm. Future use of this model will permit the evaluation of different antinociceptive strategies and help in the elucidation of new targets for the treatment of neuropathic pain.

## 1. Introduction

In addition to having to cope with the debilitating locomotor and autonomic consequences of spinal cord injury (SCI), the majority of individuals also develop chronic pain syndromes, regardless of the severity of the injury or the degree of motor recovery achieved [1–3]. Neuropathic pain after SCI is mainly characterized by severe allodynia, which is a heightened response to a stimulus, either mechanical or thermal, that would otherwise evoke little or no pain [4, 5]. In experimental SCI models, structural and chemical changes within the injured spinal cord lead to the development of behavioral hypersensitivity as characterized by the appearance of mechanical allodynia and thermal hyperalgesia on dermatomes at, above, and/or below the level of injury [4, 6]. Although these symptoms have been shown to arise from neuronal hyperexcitability in the dorsal horn, resulting from enhanced excitatory amino acid (EAA) signaling [7, 8] and/or loss of central inhibition [9], the underlying mechanisms responsible for these sensory changes remain poorly understood. Further elucidation of the signaling intermediaries involved in this altered neurotransmission after SCI is critical for the identification of putative therapeutic targets for the treatment of neuropathic pain.

Nociceptive signals are carried by both myelinated A*δ* and unmyelinated C fibers in the spinal cord. After SCI, there is an observed increase in levels of glutamate and aspartate, EAAs responsible for mediating excitatory impulses via ligand-gated ionotropic receptors, including NMDA and AMPA [7, 8]. Upregulation of AMPA receptors, which are responsible for gating the majority of fast synaptic transmission in the central nervous system (CNS), creates a hyperexcitable state in the spinal cord below the site of injury [10–13]. Blocking glutamate receptors below the injury, such as GluR3, a subunit of the AMPA receptor family, has been shown to decrease the hyperexcitability of dorsal horn neurons and lessen mechanical allodynia in rats with severe contusive SCI [14, 15]. Concomitantly with an increased activation of excitatory neurotransmitter receptors after SCI, there is a decline in inputs from inhibitory neurotransmitters such as GABA and glycine [16–18]. GABA is the primary inhibitory neurotransmitter in the spinal cord and it plays an antinociceptive role under both normal and pathological conditions [19]. Reduced GABAnergic tone after a peripheral nerve or a spinal cord injury disinhibits excitatory impulses and lowers nociceptive thresholds [20–22]. When GABA receptors are stimulated in noninjured rats using GABA_A_ and GABA_B_ receptor agonists, animals show reduced mechanical allodynia [23–25]. Glutamate decarboxylase (GAD) is a GABA synthesizing enzyme that is found in two forms, GAD_65_ and GAD_67_, within the axonal bouton and the cell body, respectively [26, 27]. There is evidence that dysregulation of these two enzymes occurs in the dorsal horn of the spinal cord after an injury [22, 28]. Glycinergic signaling is also reduced under neuroinflammatory conditions, including SCI, with an inhibition of glycine receptors, such as glycine receptor *α*3 (GLRA3) [16, 29, 30]. Inflammation increases the expression of the enzyme cyclooxygenase-2 (COX-2) and the production of prostaglandin E_2_ (PGE_2_), a GLRA3 antagonist. PGE_2_ blocks GLRA3 via a protein kinase A (PKA) pathway, making these receptors unresponsive to the inhibitory effects of glycine [31, 32]. Overall a lack of central inhibition from reduced levels of GAD_65/67_ and the muted state of GLRA3 and increased excitatory activity, via the upregulation of GluR3, are the main contributors to a hypersensitive state to peripheral stimuli after SCI [16].

The current study sought to explore the plasticity of the sensory system in a clinically-relevant SCI model with the goal of elucidating new pharmacological targets for the treatment of chronic pain. The MASCIS (25.0 mm weight drop) thoracic T8 contusion model produces a highly reproducible manifestation of persistent behavioral hypersensitivity that has been previously characterized [33–35] and is similar to the symptoms observed in a high percentage of humans after SCI [36, 37]. Rather than focus on the dysregulation of inhibitory (GAD_65/67_ and GLRA3) and excitatory (GluR3) neurotransmission within the dorsal horn, near to the injury, in the present work the temporal (1 and 12 weeks) and spatial expression profiles of these markers were investigated along the thoracic and lumbar cord. These biochemical changes were examined in relation to the development of allodynic and hyperalgesic responses on the paw and torso dermatomes. The current study provides new insight into the dysregulation of neurotransmission following SCI and the development of neuropathic pain, providing novel targets that could be used for the design of treatments for chronic pain clinically.

## 2. Materials and Methods

### 2.1. Animals

Adult female Fischer rats (Harlan Co., *n* = 24; 180–200 g) were housed according to NIH standards and The Guide for the Care and Use of Animals. The Institutional Animal Care and Use Committee of the University of Miami approved all animal procedures. Prior to surgery, rats were anesthetized intraperitoneally (45 mg/kg ketamine and 5 mg/kg xylazine) and a reflex test was performed to assess that an adequate level of anesthesia had been attained. Lacrilube ophthalmic ointment (Allergan Pharmaceuticals, Irvine, CA) was applied to the eyes to prevent drying. During surgery, the animals were kept on a homeothermic blanket system (Harvard Apparatus Ltd., Kent, England) to maintain the body temperature at 37 ± 0.5°C as measured by rectal probe.

### 2.2. Contusive Spinal Cord Injury

A moderate contusion injury was induced by the weight drop device developed at New York University [33]. Following anesthesia, a vertical incision was made along the thoracic vertebra and the superficial muscle and skin retracted. A laminectomy was performed at the thoracic vertebra T8 to expose the dorsal surface of the spinal cord underneath (T9) without disrupting the dura mater. Stabilization clamps were placed around the vertebrae at T6 and T12 to support the column during impact. The exposed spinal cord was moderately injured by dropping a 10.0 g rod from a height of 25.0 mm. The contusion impact height, velocity and compression were monitored. Animals (*n* = 2) were excluded immediately when height or velocity errors exceeded 7% or if the compression distance was not within the range of 1.75–2.25 mm. After injury, the muscles were sutured in layers and the skin closed with metal wound clips. The animals were allowed to recover in a warmed cage with water and food easily accessible. Postoperative care, including the administration of antibiotics, analgesics, and fluids, was performed as described elsewhere [38, 39]. Survival times were one week and twelve weeks after injury for the assessment of acute and chronic protein levels of GluR3, GAD_65/67_, and GLRA3 above (T5/6) and below (T10/11, L2, and L5) the injury site. Animals were randomly placed into four groups for further analyses: (i) acute noninjured (*n* = 6), (ii) chronic noninjured (*n* = 6), (iii) acute SCI only (*n* = 6), and (iv) chronic SCI only (*n* = 6).

### 2.3. Behavioral Testing

#### 2.3.1. Cutaneous Allodynia Evaluation

The hind limbs and back of the rats were tested for cutaneous allodynia using an electronic von Frey anesthesiometer (IITC Life Science Inc., Woodland Hills, CA). The animals were acclimatized to human handling by placing them in a clear Plexiglas testing apparatus (8 × 8 × 18 cm) for 4 hours daily for 14 days prior to testing. Preoperative testing began 1 week before injury to establish both individual and group baseline behaviors. After injury, tests were performed at weekly intervals for twelve weeks. Before testing, animals were allowed a 30-minute acclimation period to the apparatus. Once acclimatized, the device tip was applied perpendicular to the specific places on the ventral midplantar glabrous skin of both hind paws and on the dorsal skin dermatomes (caudal, rostral, and at level of the injury) and depressed slowly until the animal elicited a response from pressure. The value, in grams, was recorded for each of the 3 trials. A single trial of stimuli consisted of three to four applications of the von Frey tip within a 10-second period, to ensure a consistent response. The values obtained for each hind paw were averaged and the standard error of the mean (SEM) calculated. Responses to the stimulus included withdrawal, head turning, and postural changes in normal rats.

#### 2.3.2. Thermal Hyperalgesia Assessment

Paw-withdrawal response to a noxious thermal stimulus was assessed using a Hargreaves device [40]. The animals were first placed in a clear Plexiglas box mounted on an elevated Plexiglass floor. Animals were then allowed to acclimatize for approximately 30 minutes. A radiant heat source with a constant-intensity (i.e., 2.5 V) was aimed individually at the ventral midplantar surface of each one of the animal's hind paws. The time, in seconds, from initial heat source activation (normally noxious stimulus) until paw withdrawal, was recorded. A five-minute rest period was allowed between successive stimulation. Four latency measurements for each hind paw were recorded at weekly sessions and the mean and the SEM were calculated for each hind paw.

### 2.4. Biochemistry

#### 2.4.1. Western Blot Analysis

Acute and chronic protein expression levels of the GABA-synthesizing enzymes GAD_65/67_, the glycine receptor GLRA3, and the AMPA receptor GluR3 were detected by immunoblotting as described previously [36]. Blocks of spinal cord (3 mm in length) taken immediately above the injury (T5-6), immediately below the injury (T10-11), and from the lumbar cord (L2 and L5), one week and twelve weeks after injury were evaluated. Equal amounts of spinal cord lysates were added to 5x SDS-PAGE sample buffer (0.225 M Tris-Cl, pH 6.8, 50% glycerol, 5% SDS, 0.05% bromophenol blue, and 0.25 M dithiothreitol). The mixture was heated for 5 minutes at 95°C then centrifuged for 5 minutes. The samples were loaded into a polyacrylamide gel (Lonza PAGEr Gold Gels 10–20%) in equal amounts (10 *μ*L per lane). Proteins were separated using SDS-page gel electrophoresis and transferred to polyvinylidene fluoride (PVDF) membranes at 100 V for 90 minutes in transfer buffer (25 mM Tris base, 150 mM glycine, and 20% methanol). The PVDF membranes were blocked overnight in blocking solution (2% Amersham ECL blocking agent in TBS-Tween). The following day, they were incubated with antibodies against GAD_65/67_ (1 : 50,000), GluR3 (1 : 10,000), and GLRA3 (1 : 10,000) diluted in blocking solution for 60 minutes and washed four times in TBS-Tween for 10 minutes. After washing, the membranes were incubated with HRP-conjugated goat anti-rabbit IgG (1 : 10,000; Santa Cruz) for 60 minutes and washed again four times in TBS-Tween for 10 minutes. The membranes were treated with the Western Lightning Ultra Extreme Sensitivity Chemiluminescence reagent (PerkinElmer) for antibody binding detection. Quantification of bands corresponding to changes in protein levels was made using scanned densitometric analysis and the NIH Image Program 1.62f.

### 2.5. Data and Statistical Analysis

All analyses were conducted upon the completion of behavioral testing or histological preparation by an individual blinded to the animal's treatment. For statistical analyses of cutaneous allodynia and thermal hyperalgesia data, hind paw values were compared using a one-way ANOVA with a Bonferroni posttest and skin values were compared using repeated measures ANOVA with Bonferroni posttest. The density of immunoblot bands in different spinal cord regions was compared independently among the groups by one-way ANOVA with a Bonferroni posttest. Statistical significance was accepted at *P* < 0.05.

## 3. Results

### 3.1. Moderate Contusive SCI Produces Persistent Cutaneous Allodynia and Thermal Hyperalgesia

**Figure 1:**
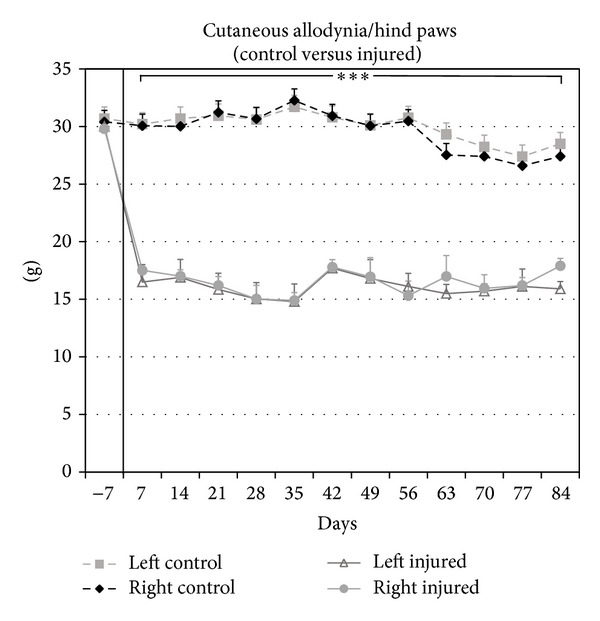
Significant increases in cutaneous allodynia on the hind paws were observed in the injured group when compared to noninjured controls. Compared to preinjury, baseline, and uninjured control withdrawal thresholds, the withdrawal responses for the injured group after SCI were significantly lower from 2 to 12 wks after SCI. Similar values were observed up to 12 wks after injury.

To measure behavioral hypersensitivity on the paw and torso dermatomes, cutaneous allodynia with definite responses indicative of pain and thermal hyperalgesia were evaluated at 1 week before injury, as a baseline, and weekly after SCI for 12 weeks. For baseline recordings, the average pressure required to elicit a painful withdrawal response of both hind paws was 30.7 ± 0.8 g. In the noninjured group, subsequent recordings over 12 weeks did not vary significantly from this baseline value (*P* > 0.05). In contrast, following SCI, a pronounced reduction in the pressure required to elicit a painful response with the von Frey filament was observed, with average withdrawal pressures of 17.3 ± 1.6 g (*P* < 0.001) and 16.3 ± 0.7 g (*P* < 0.001) at weeks 2 and 12, respectively (Figure 1).

**Figure 2:**
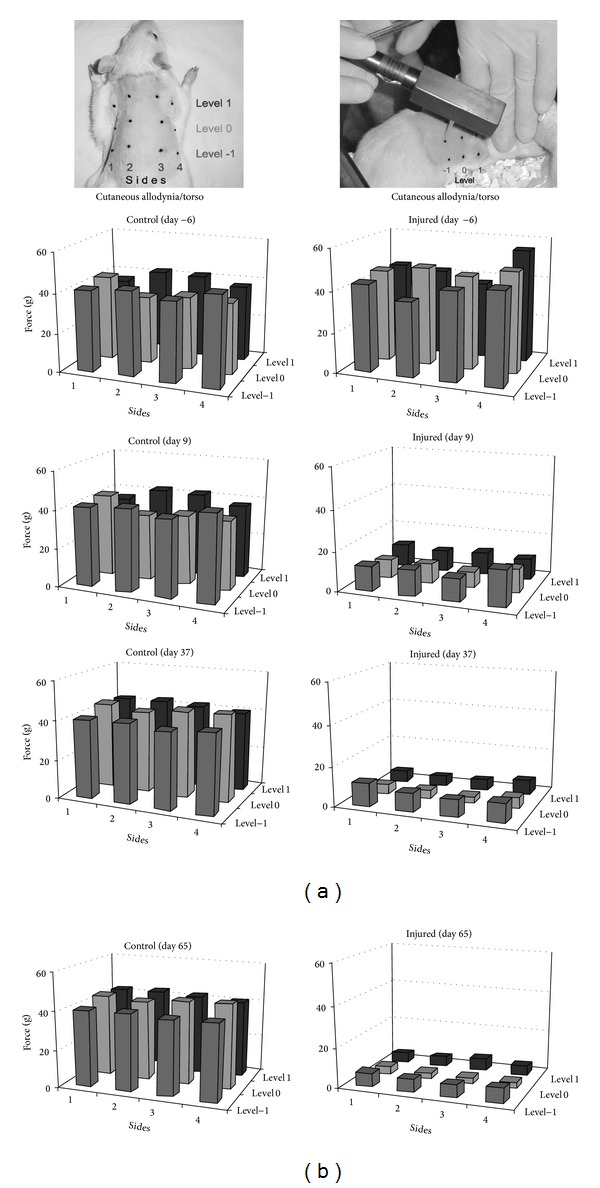
Manifestation of allodynia on the cutaneous surface of the back over time after contusive SCI. Before injury, the force required to elicit a response was equal at all points. Within 9 days after the contusion, the force was reduced 4-5-fold in all areas, with the epicenter (level 0) showing the greatest hypersensitivity. This same pattern persisted up to 90 days after SCI. Overall, a 10-fold increase in tactile allodynia was observed on the back of the animals with SCI. The data are expressed as means.

Upon the torso, cutaneous allodynia was evaluated at three different levels: above the injury (level +1), at the injury level (level 0), and below the injury (level −1), at points along the midline and laterally on both sides of the back as illustrated in Figure 2. Compared to uninjured controls, in which no significant changes were observed on torso dermatomes for the 13 week duration of the study, a pronounced and persistent reduction in the pressure required to elicit a painful response was noted after SCI at, above, and below the level of injury from week 2 to endpoint. At 2 weeks after SCI, the pressure required to elicit a painful response had decreased by up to 78.6% (*P* < 0.001), 79.1% (*P* < 0.001), and 72.8% (*P* < 0.001), respectively, at levels +1, 0, and −1 compared to baseline values (Figure 2). This pronounced allodynia persisted through 12 weeks with endpoint pressure reductions of 85.7% (*P* < 0.001), 87.2% (*P* < 0.001), and 81.2% (*P* < 0.001), respectively, at levels +1, 0, and −1 compared to baseline values.

**Figure 3:**
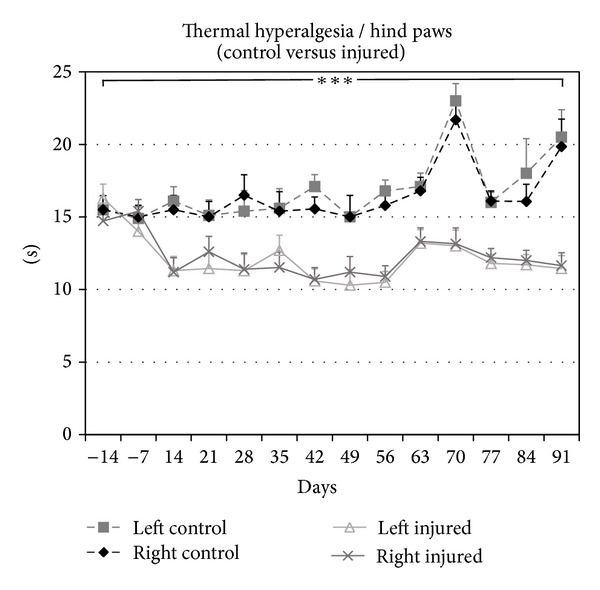
Thermal hyperalgesia developed in both hind paws, as evidenced by a decrease in paw withdrawal thresholds. At 2 weeks after injury, the withdrawal responses for both hind paws were significantly lower than the baseline withdrawal threshold, which was measured at 1 week before SCI. Similar values were observed up to 12 wks. Compared to preinjury, baseline, and uninjured control withdrawal thresholds, after SCI the withdrawal responses for the injured group was significantly lower from 2 to 12 wks after SCI. Similar values were observed up to 12 wks after injury.

The evaluation of thermal hyperalgesia on the hind paws of uninjured animals provided baseline withdrawal values of 14.7 ± 0.91 s from the heat source. Two weeks after SCI, animals exhibited a 23.4% increase in withdrawal times (11.3 ± 1.1 s, *P* < 0.001) compared to baseline values, indicating a greater sensitivity to the noxious thermal stimulus (Figure 3). The degree of hyperalgesia on the hind paws persisted through endpoint, with a 30.9% increase in withdrawal times at 12 weeks after SCI (11.6 ± 0.8 s, *P* < 0.001), compared to uninjured recordings.

### 3.2. Increases in Markers of Glutamatergic Neurotransmission and Decreases in Glycinergic and GABAnergic Signaling Are Observed after Contusive SCI

GluR3, GAD_65/67_, and GLRA3 levels were evaluated by immunoblotting in spinal cord samples taken from above (T5/6) and below (T10/11, L2, and L5) the injury site. Protein production was assessed at 1 week (acute) or 12 weeks (chronic) after SCI for comparison to analogous regions taken from uninjured controls.

**Figure 4:**
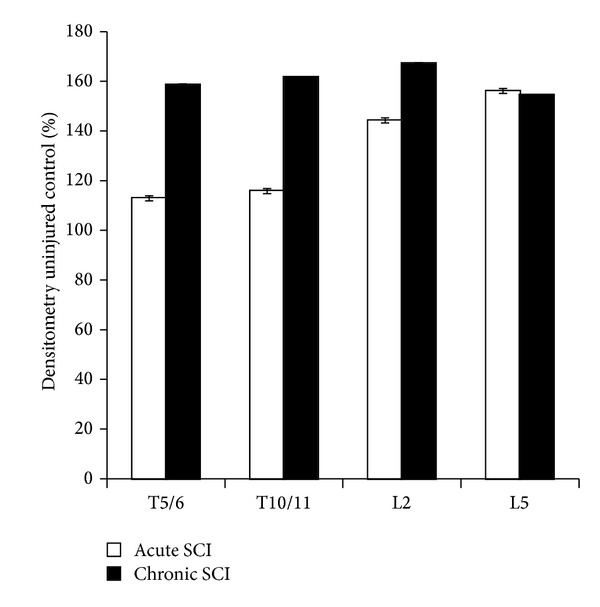
Protein expression of the AMPA receptor GluR3 as detected by immunoblotting. At both acute (1 week) and chronic (12 wks) time points, the protein expression of GluR3 increased at all levels of the spinal cord, but most notably at the lumbar level. This increase was even more notable on week 12, at T5/6, T10/11, and L2, however; the expression of GluR3 at L5 did not change significantly from week 1.

Expression of the AMPA receptor GluR3, for the excitatory neurotransmitter glutamate, showed increases both acutely and chronically after SCI at all levels of the spinal cord (Figure 4). Above the level of SCI (T5/6), GluR3 expression increased 12.9% (*P* < 0.001) and 58.6% (*P* < 0.001) at 1 and 12 weeks after injury, respectively. Similar increases in GluR3 expression were observed at T10/11 level, 15.8% (*P* < 0.001) and 61.7% (*P* < 0.001), respectively, at 1 and 12 weeks after SCI. Below the level of injury (at L2 and L5), GluR3 expression increased significantly and sooner after SCI and at 1 week (L2, 44.2% increase, *P* < 0.001; L5, 56.1% increase, *P* < 0.001) and 12 weeks (L2, 67.2% increase, *P* < 0.001; L5, 54.5% increase, *P* < 0.001) after injury compared to uninjured controls.

**Figure 5:**
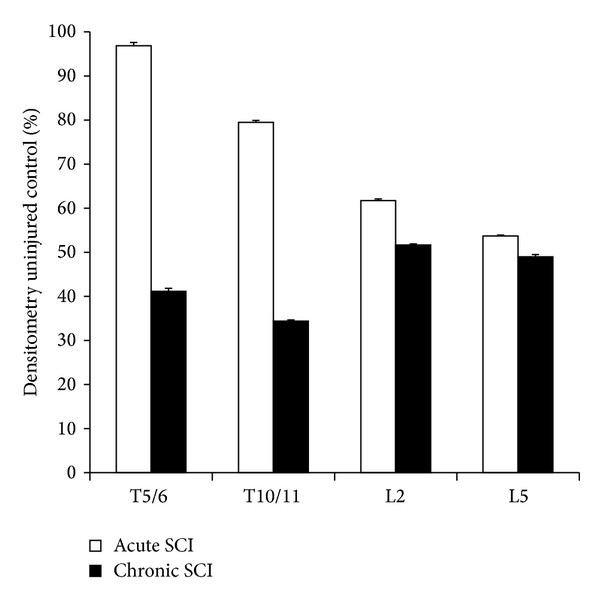
Protein expression of the GABA synthesizing enzyme GAD_65/67_ as detected by immunoblotting. There was a decrease in GAD_65/67_ expression at all levels, both in the acute and chronic paradigms. However, the downregulation of GAD_65/67_ was most noticeable at 12 wks. In the earlier time point, especially above the injury site (T5/6), the expression of GAD_65/67_ decreased slightly but continued a steady decline at all of the other levels.

GABAnergic signaling was assessed using immunoblot detection of the GABA-synthesizing enzymes GAD_65/67_ (Figure 5). Although significant reductions in GAD_65/67_ were observed at all levels of the spinal cord at 12 weeks after SCI, at 1 week after injury no changes were observed above the level of SCI, only below the injury site. Above the level of SCI, there was a 58.9% reduction in GAD_65/67_ (*P* < 0.001) at 12 weeks, but not 1 week after injury compared to uninjured controls. Decreased GAD_65/67_ expression was also observed at the T10/11 level, 20.5% (*P* < 0.001) and 65.6% (*P* < 0.001), respectively, at 1 and 12 weeks after SCI. Below the level of injury (at L2 and L5), GAD_65/67_ expression decreased more significantly and sooner after SCI than levels detected above the injury site and at 1 week (L2, 38.3% decrease, *P* < 0.001; L5, 46.3% decrease, *P* < 0.001) and 12 weeks (L2, 48.3% decrease, *P* < 0.001; L5, 51.1% decrease, *P* < 0.001) after injury compared to uninjured controls.

**Figure 6:**
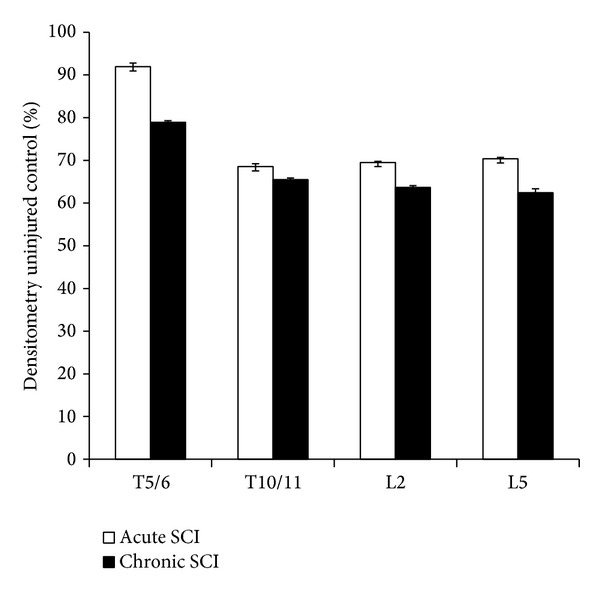
Protein expression of the glycine receptor GLRA3 as detected by immunoblotting. At 1 week as well as at 12 wks, the protein expression of GLRA3 had downregulated at all levels of the spinal cord, but most notably below the injury site. Acutely, GLRA3 values showed only a small decrease at T5/6. However, this downregulation became more apparent at the other three levels. The same pattern was seen in the chronic paradigm.

The glycine receptor, GLRA3, was used as a marker for glycinergic signaling. Like GAD_65/67_, GLRA3 showed pronounced and persistent decreases in expression along the spinal cord after SCI (Figure 6). Above the level of SCI, there was a 21.1% reduction in GLRA3 (*P* < 0.001) at 12 weeks after injury but no change at 1 week compared to uninjured controls. Decreased GLRA3 expression was also observed at T10/11 level, 31.1% (*P* < 0.001) and 34.2% (*P* < 0.001), respectively, at 1 and 12 weeks after SCI. Below the level of injury (at L2 and L5), GLRA3 expression decreased significantly and sooner after SCI than levels detected above the injury site and at 1 week (L2, 30.5% decrease, *P* < 0.001; L5, 29.6% decrease, *P* < 0.001) and 12 weeks (L2, 36.4% decrease, *P* < 0.001; L5, 37.6% decrease, *P* < 0.001) after injury compared to uninjured controls.

## 4. Discussion

Neuropathic pain is a major secondary complication after SCI. Approximately 65% of SCI individuals report hypersensitivity at or below level of injury, and 30% of those individuals classify the pain as excruciating [2, 3, 41, 42]. The present study used a clinically-relevant, moderate contusion SCI model in the adult rat to investigate the behavioral sequelae and main molecular mechanisms that underlie the manifestation of central pain after SCI. Several groups have shown previously that SCI-related neuropathic pain is due, in part, to an increase in excitability in the CNS parallel to a decrease in inhibitory inputs from the spinal dorsal horn [6, 18, 42]. The summation of these two events leads to a hyperexcitable state in the CNS and the emergence of pain syndromes [37]. Temporal and spatial behavioral and molecular data from the present study supports the hypothesis that reductions in inhibitory GABAnergic and glycinergic signaling, via GAD_65/67_ and GLRA3, and increased excitatory glutamatergic signaling, via GluR3, are involved in neuronal hyperexcitability and the ensuing manifestation of neuropathic pain following SCI.

GABA and glycine are the two major molecules involved in fast inhibitory transmission in the CNS [43, 44]. GABAnergic and glycinergic fibers descend from supraspinal sites and innervate the dorsal horn directly. Their role in sensory processing occurs through post and presynaptic interaction of GABAnergic and glycinergic dorsal horn neurons and interneurons with other nearby neurons, as well as with spinal sensory axon terminals [18]. GABA is synthesized in the neurons from glutamic acid by the enzyme GAD (with two isoforms GAD_65/67_). GABAnergic neuronal cell bodies can be found in the grey matter, particularly in the superficial laminae I–III of the dorsal horn, where they receive input from myelinated A*δ* and unmyelinated C-fibers and thus help regulate nociceptive transmission [45–48]. Trauma to the spinal cord triggers several cascades of neurochemical events at the local level that cause changes in pain perception throughout the nervous system. The initial signal may possibly come from damaged fibers in the dorsal horn, which then activate microglia to release the neurotrophic factor BDNF. BDNF in turn binds to trkB receptors, causing the downregulation of the potassium chloride exporter KCC2 and the depolarization of dorsal horn neurons [18]. The intracellular chloride ion gradient is controlled synergistically by neurons and glial cells, which modulate chloride transporters as well as GABA and glutamate concentrations in order to maintain a proper balance of chloride ions [21]. However, once the expression of KCC2 is reduced, chlorine homeostasis is altered leading to a decrease in GABAnergic inhibitory transmission, which results in sensory hypersensitivity [6, 47]. Injury also causes the loss of GABAnergic neurons and interneurons in the spinal superficial dorsal horn through caspase-3 activation, in addition to a downregulation in the expression of GAD_65/67_, especially within laminae I–III of the dorsal horn [47]. However, the exact mechanisms underlying the downregulation of GAD_65/67_ have not yet been fully elucidated. One hypothesis put forth by Gwak and Hulsebosch is that the upregulation of glutamate following SCI, combined with an increase of other pain promoting molecules released by glial cells, such as ATP, IL-1, IL-6, TNF, NO, and ROS, triggers intracellular downstream cascades that affect GAD gene expression [21].

Glycine is a ubiquitous amino acid and it is the second most important fast inhibitory neurotransmitter in the CNS [43, 49]. Glycinergic neurons are localized throughout the spinal grey matter, overlapping with GABAnergic neurons, although they are more localized to deeper layers of the dorsal horn (laminae III-IV) [46]. Neurons in deeper dorsal horn laminae can receive inputs from GABAnergic and glycinergic synapses at the same time, since both neurotransmitters can be released concomitantly [50–53]. As for the glycine receptors, most are heteromeric and contain four *α* subunits (GLRA1-4) and one *β* subunit, but in the adult nervous system subunit *α*1 is the most abundant. Our experiment focuses on the glycine receptors containing subunit *α*3 (GLRA3), since it has been established that they are involved in inflammatory responses. GLRA3 is not a widely expressed subunit, but it can be found in the more superficial layers of the dorsal horn where nociceptive A*δ*- and C-fiber afferents terminate [54].

Chronic inflammatory pain after SCI is marked not only by a decrease in GABAnergic tone in the dorsal horn, as discussed above, but also by a decline in glycinergic inhibition. Fast inhibition in the spinal cord by both GABA and glycine occurs mainly in presynaptic terminals [55]. However, glycine is the main component responsible for the postsynaptic transmission occurring in superficial levels of the adult dorsal horn [32, 54]. The inhibition of this type of glycinergic transmission is thought to be regulated by the inflammatory mediator prostaglandin E_2_ (PGE_2_), which is mainly localized in the superficial layers of the dorsal horn where most terminals for nociceptive fibers are found. Peripheral inflammation causes an upregulation in the production of PGE_2_ by COX-2 and prostaglandin E synthase in the spinal cord [56]. In turn, postsynaptic PGE receptors of the EP2 subtype are activated and trigger a G-protein coupled cascade that increases intracellular levels of cyclic adenosine monophosphate (cyclic AMP). Protein kinase A (PKA), which is activated by cyclic AMP, goes on to phosphorylate and inhibit glycine receptors containing the *α*3 subunit [30, 32, 54]. Studies using rodent models have shown that the inhibition of glycinergic transmission in the superficial layers of the dorsal horn contributes to both mechanical allodynia and thermal hyperalgesia [57–59]. Thus, increasing the inhibitory tone in the CNS through the modulation of the glycine receptor subtype GLRA3 is a potential pharmacological target for the treatment of neuropathic pain after SCI.

Glutamate is the major excitatory neurotransmitter in the CNS and, as such, it is a crucial component for mediating excitatory synaptic transmission [60]. There are several receptors for glutamate, but it has been well established that the AMPA receptor subtype plays a powerful role in fast excitatory currents and, consequently, in the stimulation of pain behavior [61–63]. Most AMPA receptors are heterotetrameric structures with four subunits (GluR1-4). In 2008, Polgár and colleagues studied the localization of AMPA receptors in the adult rat spinal cord and concluded that most glutamatergic synapses in laminae I–III of the dorsal horn contain AMPA receptors [62]. GluR1 is the most abundant subtype in laminae I and II where it overlaps with GluR2 and/or GluR3, while GluR4 is confined to some lamina I projection cells. In the present investigation we focused on the expression of the less studied GluR3 after SCI. The combination of diminished inhibitory inputs from dorsal horn neurons along with a rise in the excitatory tone driven by the neurotransmitter glutamate leads to a chronic hypersensitive state. Gwak and Hulsebosch have shown that after injury there is a prolonged activation of dorsal horn neurons due to an increase in glutamate-driven excitation [21]. In adult rats, the great majority of glutamatergic synapses in laminae I–III have glutamate receptors of the AMPA type, which mediate fast excitatory transmission in the dorsal horn [62, 64]. Polgár and colleagues studied the localization of the four AMPA receptor subtypes and found that between 57 and 65% of the puncta in laminae I–II and 80% in lamina III were positive for GluR3 [62]. Their findings along with data from Tong and Macdermott [12] showed that neurons expressing AMPA receptors receive primary afferent inputs associated with pain perception, making them crucial players in the synaptic transmission between peripheral nociceptive neurons and spinal cord neurons. Similar to the upregulation in the production of PGE_2_ following peripheral inflammation described previously, noxious peripheral nervous system (PNS) stimulation also triggers the release of glutamate from central terminals of primary afferents into the spinal cord [65]. In turn, glutamate activates AMPA receptors in the dorsal horn via several protein kinase cascades that phosphorylate these receptors, causing an enhanced response to painful stimuli [13, 66]. In a rat model of diabetic neuropathy described by Tomiyama et al. [65], there was an increase in the levels of mRNAs coding for GluR1, GluR2, and GluR3 in all layers of the dorsal horn of experimental animals. A great number of studies have established the role of AMPA receptors in the development of chronic pain, which reinforces their potential as targets for novel analgesic compounds.

As we have discussed, several molecular mechanisms in the spinal cord are involved in the sensitization of nociceptive inputs. Our study focused on the relative temporal and spatial changes that occur in the expression of two major inhibitory transmitters, GABA and glycine, and of the main excitatory transmitter in the nervous system, glutamate, after a mechanical trauma to the adult rat spinal cord. Our results support previous findings that the loss of inhibitory tone from dorsal horn neurons coupled with an increase in motoneuron excitability is one of the main molecular pathways implicated in chronic neuropathic pain following SCI [21, 42, 67] and that targeting both these systems may offer more effective therapeutics for ameliorating neuropathic pain after SCI [61]. It is important to reiterate that our immunoblotting results indicated that GluR3 levels were upregulated while GLRA3 expression was downregulated more notably below the level of lesion at the acute time point. This observation is also in accordance to several human studies that have shown that most SCI-related pain syndromes are reported below the injury level. This particular type of chronic pain that is associated with SCI is termed “below-level central pain” [9, 41, 68, 69]. All three neurotransmitters explored in the current study are major components of the nervous system and, as such, have been extensively characterized in the literature. In turn, the focus on their receptors and their potential role as therapeutic drug agents for the treatment of neuropathic pain has increased significantly in the pharmacology field. It is our goal to expand the understanding of these underlying mechanisms, especially in the context of a clinically-relevant SCI model, so that new targets can be identified as early treatment options for the management of neuropathic pain.

## 5. Conclusions

The current study demonstrates that adult rats with a thoracic 25.0 mm NYU contusion exhibit behavioral hypersensitivity relevant to human SCI, determined by cutaneous allodynia and thermal hyperalgesia development, combined with a loss of central inhibition and enhanced excitability due to molecular changes in GluR3, GAD_65/67_ and GLRA3. Future use of this model will permit us to evaluate how different anti-nociceptive or regeneration strategies might alter sensory responses and help in the elucidation of new targets for the treatment of neuropathic pain.

## Abbreviations

SCI: Spinal cord injury
GABA: Gamma-aminobutyric acid
EAA: Enhanced excitatory amino acid
NMDA: N-Methyl-D-aspartate
AMPA: 2-Amino-3-(5-methyl-3-oxo-1,2-oxazol-4-yl) propanoic acid
GluR: Glutamate receptor
GAD_65/67_: Glutamate decarboxylase 65/67
GLRA3: Glycine receptor *α*3
COX-2: Cylooxygenase-2
PGE_2_: Prostaglandin E_2_
PKA: Protein kinase A
CNS: Central nervous system
PNS: Peripheral nervous system
BDNF: Brain-derived neurotrophic factor
trkB: Tropomyosin-related kinase B
KCC2: Potassium chloride cotransporter 2
ATP: Adenosine-5′-triphosphat;
IL-1: Interleukin-1
IL-6: Interleukin-6
TNF: Tumor necrosis factor
NO: Nitric oxide
ROS: Reactive oxygen species
cyclic AMP: Cyclic adenosine monophosphate
TBS: Tris buffered saline
PDVF: Polyvinylidene fluoride
ECL: Enhanced chemiluminescence
HRP: Horseradish peroxidase
IgG: Immunoglobulin G
NIH: National Institutes of Health
ANOVA: Analysis of variance
SEM: Standard error of the mean
